# Molecular Crosstalk Between MYC and HIF in Cancer

**DOI:** 10.3389/fcell.2020.590576

**Published:** 2020-11-05

**Authors:** Yanping Li, Xiao-Xin Sun, David Z. Qian, Mu-Shui Dai

**Affiliations:** ^1^Department of Molecular and Medical Genetics, School of Medicine, Portland, OR, United States; ^2^The OHSU Knight Cancer Institute, Oregon Health and Science University, Portland, OR, United States

**Keywords:** MYC, HIF1α, HIF2α, transcription, protein stability, metabolism

## Abstract

The transcription factor c-MYC (MYC thereafter) is a global regulator of gene expression. It is overexpressed or deregulated in human cancers of diverse origins and plays a key role in the development of cancers. Hypoxia-inducible factors (HIFs), a central regulator for cells to adapt to low cellular oxygen levels, is also often overexpressed and activated in many human cancers. HIF mediates the primary transcriptional response of a wide range of genes in response to hypoxia. Earlier studies focused on the inhibition of MYC by HIF during hypoxia, when MYC is expressed at physiological level, to help cells survive under low oxygen conditions. Emerging evidence suggests that MYC and HIF also cooperate to promote cancer cell growth and progression. This review will summarize the current understanding of the complex molecular interplay between MYC and HIF.

## Introduction

Cancer cells undergo significant metabolic changes to sustain the rapid cell proliferation, adapt to environmental challenges such as hypoxia, and promote invasion and metastasis. While HIF signaling is essential for normal cell adaptation to oxygen homeostasis, it also plays a key role in the growth of solid tumors, which inevitably contain poorly vascularized regions due to rapid tumor cell proliferation (Brahimi-Horn and Pouyssegur, 2009; Rankin and Giaccia, 2016; Lee et al., 2020). Tumor hypoxia is typically correlated with more aggressive phenotype and poor prognosis partly due to its contribution to therapeutic resistance and tumor cell invasion and metastasis via activating various cell survival pathways. MYC signaling also plays pivotal roles in regulating cancer cell metabolism and vasculogenesis (Baudino et al., 2002; Dang, 2012b; Stine et al., 2015). In this review, we focus on the current understanding of the molecular interplay between MYC and HIF in cancer cell metabolism, growth and progression as well as its potential implication in cancer therapy.

**FIGURE 1:**
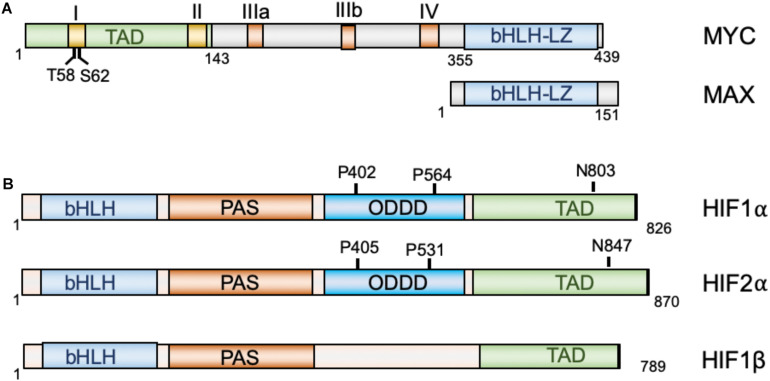
Diagrams of MYC and MAX **(A)** and HIF proteins **(B)**. MYC and Max dimerize via their bHLH-LZ domains, whereas HIF1α and HIF2α dimerize with HIF1β via their N-terminal bHLH domains. TAD, transactivation domain; bHLH, basic helix-loop-helix; LZ, leucine zipper; PAS, Per-Arnt-Sim homology; ODDD, oxygen-dependent degradation domain.

The MYC oncoprotein is a master regulator of transcription that activates or represses gene expression to coordinate diverse cellular processes, including cellular division, differentiation, apoptosis, angiogenesis, DNA replication, RNA processing, metabolism, and ribosome biogenesis (Bretones et al., 2015; Kress et al., 2015; Baluapuri et al., 2020). MYC heterodimerizes with the MAX protein, via its C-terminal basic helix-loop-helix-leucine zipper (bHLH-LZ) domain, to bind to the consensus CACGTG (E-box) elements on chromatin. At the N-terminus of MYC lies its transactivation domain (TAD) that contains the conserved Myc box I (MBI) and MBII essential for both transcriptional activation and repression (Farrell and Sears, 2014; Tu et al., 2015; Baluapuri et al., 2020). Two important phosphorylation sites, Threonine 58 (T58) and Serine 62 (S62), within MBI are critical for the regulation of MYC stability and activity in response to cell growth signals (Farrell and Sears, 2014; Chen Y. et al., 2019). The central region of MYC contains the MBIIIa, MBIIIb, and MBIV which are important for transcriptional activity and pro-apoptotic activity (Cowling et al., 2006; Kurland and Tansey, 2008; Thomas et al., 2015, 2016). For example, MBIIIa interacts with the histone deacetylase HDAC3 to suppress transcription and MBIIIb associates with WDR5 to facilitate H3K4 methylation and MYC recruitment to chromatin (Thomas et al., 2015), whereas MBIV association with transcriptional coregulator host cell factor-1 (HCF-1) is critical for MYC-driven tumorigenesis (Thomas et al., 2016; Figure 1A). Recent genome-wide and gene specific studies have revealed several emerging models for MYC function, including specific-gene regulation, global gene activation, and gene-specific affinity models (Kress et al., 2015; Baluapuri et al., 2020). Together, MYC controls the expression of genes involved in almost all aspects of tumor hallmarks. Consistent with its roles in oncogenesis, MYC is frequently overexpressed in human cancers via various mechanisms, including gene amplification, chromosomal translocation, increased MYC translation, deregulated MYC protein stabilization, or constitutive activation of upstream pathways such as Wnt, Notch, Hedgehog signaling (Nesbit et al., 1999; Dang, 2012b; Kress et al., 2015). High levels of MYC expression is associated with poor patient outcomes. Transgenic overexpression of MYC induces tumorigenesis in mice and inactivation of MYC in MYC-driven tumors causes tumor regression in multiple tumor models (Gabay et al., 2014), further demonstrating the key role for MYC in tumorigenesis.

The hypoxia-inducible factors (HIFs) are transcription factors that regulate the transcription of a large array of genes involved in metabolism, cell survival, proliferation, migration, invasion, angiogenesis, immune evasion and resistance to therapies in response to hypoxia (Brahimi-Horn and Pouyssegur, 2009; Rankin and Giaccia, 2016; Lee et al., 2020) and are recognized as the master regulators of oxygen homeostasis (Semenza, 2014). HIFs are heterodimers consisting of an oxygen-regulated α-subunit and a non-oxygen-regulated stable β subunit. HIFα consists of three isoforms: HIF1α, HIF2α, and HIF3α (Brahimi-Horn and Pouyssegur, 2009; Semenza, 2014). Both HIF α and β subunits contain a basic helix-loop-helix (bHLH) domain and the Per-Arnt-Sim homology (PAS) domain, which accounts for the dimerization between the α and β subunits (Wang et al., 1995). HIF1α and HIF2α dimerizes with HIF1β to bind to the 5′-RCGTG-3′ (*R* = A or G) core sequence of the hypoxia-response elements (HREs) on DNA to regulate gene expression (Semenza et al., 1996). The C-terminal half of HIFα contains the TAD that is critical for the recruitment of the p300/CREB-binding protein (CBP) coactivators (Arany et al., 1996). In the middle region of HIFα lies the oxygen-dependent degradation domain (ODDD), which regulates HIFα protein stability (Figure 1B). Under normoxic conditions, two proline residues at the ODDD undergo hydroxylation by prolyl-hydroxylase domain enzymes (PHD1-3) encoded by the EGLN1-3 genes (Semenza, 2014; Ivan and Kaelin, 2017; Semenza, 2020). Hydroxylation of proline residues enables recognition of HIFα by the E3 ligase, von Hippel-Lindau protein (pVHL), thereby promoting ubiquitination and proteasome-mediated degradation of HIFα (Ivan et al., 2001; Jaakkola et al., 2001; Yu et al., 2001). In addition, factor inhibiting HIF1 (FIH1) inhibits HIFα activity by hydroxylating an asparaginyl residue at the C-terminal TAD of the HIF1α and HIF2α to block the binding of p300/CBP coactivators (Lando et al., 2002a,b). PHD utilizes O_2_, ferrous iron, and α-ketoglutarate (2-OG) as substrates (Markolovic et al., 2015). In response to hypoxia, the activity of PHD is first inhibited due to lack of sufficient oxygen, resulting in HIFα escaping the hydroxylation and consequent stabilization. The enzymatic activity of FIH1 is also dependent on substrates oxygen and 2-OG and ferrous iron, and thus its activity is also inhibited in response to hypoxia. Consequently, the stabilized HIFα translocates into the nucleus, dimerizes with HIFβ and activates the transcription of hundred genes involved in cellular adaptation and survival under hypoxic conditions, including genes involved in glycolysis, erythropoietin (Epo) and VEGF, etc. While HIF1α and HIF2α cooperatively regulate genes involved in angiogenesis and metastasis (Gao et al., 2009), HIF1α preferentially induces genes involved in glycolysis (Hu et al., 2003; Wang et al., 2005) and HIF2α stimulates genes important for tumor growth and metastasis, amino acid and lipid metabolism, cell cycle progression and maintaining stem cell pluripotency (Hu et al., 2003; Covello et al., 2006; Gruber et al., 2007; Xia et al., 2009). Consistent with their role in metabolic reprogramming, angiogenesis, metastasis, therapeutic resistance (Rankin and Giaccia, 2016; Lv et al., 2017), HIFs are frequently overexpressed in various cancer cells (Zhong et al., 1999; Semenza, 2003; Soni and Padwad, 2017).

## The Regulation of MYC by HIF

In physiological normoxic conditions, MYC and HIF are expressed at low levels due to rapid protein degradation. Yet, both are essential for normal cell homeostasis and animal development. Homozygous deletion of c-MYC is embryonic lethal at E10.5 as it is essential for normal cell growth and proliferation (Davis et al., 1993). Homozygous deletion of HIF1α is also embryonic lethal at E10.5 due to defects in circulatory system and hematopoiesis (Iyer et al., 1998; Compernolle et al., 2003; Yoon et al., 2006). HIF2α null mice mostly die by E13.5 but sometimes survive only until birth due to impaired lung maturation, bradycardia, vascular defects and mitochondrial dysfunction (Tian et al., 1998; Peng et al., 2000; Compernolle et al., 2002). It is not clear whether MYC functionally interacts with HIFs in these development events.

**FIGURE 2:**
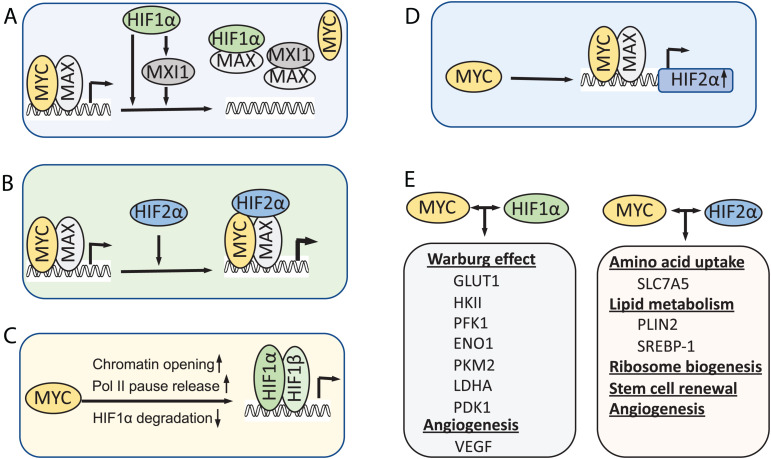
Models for the interplay between MYC and HIF. **(A)** HIF1α inhibits MYC activity under hypoxia. **(B)** HIF2α promotes MYC activity by stabilizing MYC-MAX complex. **(C)** MYC, when overexpressed, increases HIF levels and activity. **(D)** MYC also directly promotes HIF2α expression. **(E)** In cancer cells when MYC levels are high, MYC collaborates with HIFs to induce metabolic rewiring, tumor angiogenesis and CSC renewal, thereby promoting tumor cell growth and progression.

Under hypoxia, MYC activity is inhibited by HIF1α as an adaptive response that promotes cell survival under low oxygen conditions. Evidence suggests several mechanisms underlying this inhibition (Figure 2A). First, HIF1α can antagonize MYC transcriptional activity at MYC target genes by interfering with MYC binding to protein partners. HIF1α binds to MAX and disrupts MYC/MAX complexes, leading to reduced cyclin D2 expression, induction of p21, and G1-phase arrest (Gordan et al., 2007). The MAD family of proteins MAD and MXI1 compete with MYC for binding to MAX, thus inhibiting MYC activity (Conacci-Sorrell et al., 2014). HIF1-dependent induction of MXI1 under hypoxia directly represses MYC target genes that are involved in mitochondrial biogenesis, such as PGC1β (Zhang et al., 2007), or apoptosis, for example, ornithine decarboxylase (ODC) (Corn et al., 2005). Second, HIF1α was also shown to directly inhibit MYC transcriptional activity by DNA-binding site competition. It was shown that HIF1α displaces MYC binding from the p21 promoter and upregulates the expression of p21 (Koshiji et al., 2004). HIF1α also competes against MYC for binding to SP1, a known coactivator of MYC, at the promoters of MYC target genes, such as MSH2, MSH6, and NBS1, which encode DNA repair proteins (Koshiji et al., 2005; To et al., 2006), and the recently reported E−type prostanoid (EP4) receptors (Seira et al., 2018). Third, several studies showed that HIF promotes proteasomal degradation of MYC under chronic hypoxia condition (Zhang et al., 2007; Wong et al., 2013). HIF is required for the hypoxia induced degradation of MYC depending on the cell type and system used (Zhang et al., 2007; Li et al., 2009; Wong et al., 2013; Zarrabi et al., 2017). In addition, an early study also showed that HIF1α physically interacts with MYC through its N-terminus containing bHLA/PAS domains, which is sufficient to induce p21 expression and G1 arrest (Koshiji et al., 2004). It remains to be determined whether such physical interaction directly inhibits MYC activity.

In contrast to HIF1α, HIF2α has been shown to promote MYC activity. Phosphorylation of HIF2α at T324 by protein kinase D1 prevents HIF2α from competing with MYC for SP1 binding (To et al., 2006). Instead, overexpression of HIF2α enhances SP1 activity and promote MYC-driven IL-8 expression in human microvascular endothelial cells (Florczyk et al., 2011). HIF2α also enhances MYC activity in VHL-deficient clear cell renal carcinoma cells (ccRCCs) and primary mouse embryo fibroblasts (Gordan et al., 2007, 2008). Consistently, HIF2α deletion reduced MYC target transcriptome in mouse ccRCC models (Hoefflin et al., 2020). It was shown that HIF2α promotes MYC activity by stabilizing the MYC/MAX complex (Zhang et al., 2007; Xue et al., 2015; Figure 2B). Interestingly, HIF2α-induced stabilization of MYC/MAX heterodimer is much stronger than HIF1α-induced degradation of MYC in cancer cells, leading to MYC activation under hypoxia (Xue et al., 2015).

## The Regulation of HIF by MYC

Emerging evidence has indicated that MYC regulates the levels and activity of HIF1α (Figure 2C). Transient knockdown of MYC down-regulates HIF1α protein levels in multiple myeloma (MM) cells (Zhang et al., 2009). Overexpression of MYC in colon cancer and esophageal cancer cells promoted the expression of HIF1α at post-transcriptional level (Chen et al., 2013; Weili et al., 2019). Overexpression of MYC significantly stabilizes HIF1α and enhances HIF1α accumulation under both normoxic and hypoxic conditions in normal immortalized mammary epithelial cells and breast cancer cells (Doe et al., 2012). Accumulation of HIF1α by MYC leads to the induction of HIF1α targets and is required for MYC-induced anchorage-independent cell growth and proliferation (Doe et al., 2012). Mechanistically, It was shown that MYC prevents HIF1α degradation via reducing HIF1α binding to the pVHL complex, although it increases the level of pVHL complex components (Doe et al., 2012). Further, a recent study showed that MYC promotes pVHL SUMOylation while repressing its ubiquitination, thereby inhibiting HIF1α ubiquitination and proteasomal degradation (Fu et al., 2016). Besides hypoxia, HIF1α expression can be increased via oxygen-independent mechanisms under certain normoxic conditions such as reactive oxygen and nitrogen species. MYC increases mitochondrial oxidative phosphorylation (mtOXPHOS) and the generation of reactive oxygen species (ROS) (Lee et al., 2017). Increased levels of mitochondrial-generated ROS lead to the stabilization and accumulation of HIF1α by inhibiting PHDs in non-hypoxic conditions (Patten et al., 2010; Lee et al., 2017). Supporting this notion, MYC inhibits brusatol-induced HIF1α degradation by increasing mitochondrial ROS production and subsequent ROS-mediated transition of ferrous iron to ferric iron (Oh et al., 2017).

MYC may also increase HIF1α activity at the chromatin levels. HIF1α preferentially binds to transcriptionally active loci marked by the presence of histone H3 lysine 4 methylation and RNA polymerase II (Xia and Kung, 2009). MYC may promote chromatin opening by recruiting histone acetyl-transferase (HAT) and chromatin remodeling complexes (Tu et al., 2015) and facilitate RNA polymerase II pausing release (Rahl et al., 2010). MYC binding may be required for recruitment of transcriptional activators or repressor to the promoter of HIF1 target genes (Mongiardi et al., 2016). It will be interesting to examine whether MYC dynamically interplays with HIF1α at the promoter of genes that are both MYC and HIF targets, via physical interaction or sequential events at the promoters.

Recently, MYC has been shown to regulate the HIF2α expression (Figure 2D). MYC binds to HIF2α gene promoter preferentially in Sca1^+^ cancer stem cells (CSCs) in a MYC-driven mouse T-cell leukemia model and the equivalent ABCG2+ CSC population in human acute lymphoblastic lymphomas and activates HIF2α expression (Das et al., 2019). HIF2α is known to regulate stem cell function by inducing the expression of Oct4 (Covello et al., 2006) and ALKBH5, an m6A demethylase that demethylates Nanog mRNA and increases Nanog expression (Soleymani Abyaneh et al., 2018). Indeed, the stem cell factors Nanog and Sox2 facilitate MYC regulation of HIF2α, playing a critical role in stem cell renewal and tumor stemness (Das et al., 2019).

## MYC and HIF Cooperate to Reprogram Cancer Cell Metabolism and Promote Tumor Cell Growth and Progression

Cancer cells undergo metabolic switch from oxidative phosphorylation to aerobic glycolysis even under aerobic conditions, called Warburg effect (Vander Heiden et al., 2009). This metabolic rewiring is under direct management by various oncogenes, such as MYC and HIF1 (Cantor and Sabatini, 2012; Dang, 2012a,b). MYC regulates multiple stages of cell metabolism and plays a key role in cancer cell metabolic reprogramming. MYC promotes glucose flux by inducing the expression of glucose transporter1 (GLUT1) and lactate dehydrogenase A (LDHA) (Shim et al., 1997; Osthus et al., 2000); It also upregulates almost all glycolytic enzymes, including hexokinase II (HK II), phosphofructokinase (PFKM), enolase 1 (ENO1), pyruvate dehydrogenase kinase1 (PDK1), and pyruvate kinase (PKM2) (Shim et al., 1997; Osthus et al., 2000; Li et al., 2003; Kim et al., 2004; Stine et al., 2015). MYC upregulates hnRNP proteins to regulate the pyruvate kinase alternative splicing that favors aerobic glycolysis (David et al., 2010). Furthermore, MYC promotes mitochondrial biogenesis by stimulating the expression of mitochondrial transcription factor A (TFAM), a key mitochondria transcription and DNA replication factor, and many genes involved in mitochondrial structure and function (Li et al., 2005). Overall, mitochondrial function is promoted by MYC, allowing cancer cells to proliferate in oxygen and nutrient sufficient conditions. Moreover, MYC also regulates glutaminolysis (Dang et al., 2009; Stine et al., 2015), which converts glutamine to glutamate by glutaminase and then to α-ketoglutarate (α-KG) by glutamate dehydrogenase. α-KG can enter TCA cycle for producing ATP and precursors for lipid, nucleotide and amino acid biosynthesis. MYC enhances glutamine uptake and metabolism by directly stimulating the transcription of glutamine metabolism genes, such as the glutamine transporter SLC1A5/ASCT2 (Wise et al., 2008) and glutaminase 2 (GLS2) (Xiao et al., 2015). MYC also increases the expression of glutaminase 1 (GLS1) indirectly by transcriptionally repressing miR-23a/b, which targets the gene 5′-UTR (Gao et al., 2009). Consequently, deregulated MYC makes cancer cells addicted to glutamine and deprivation of glutamine causes cell death (Wise et al., 2008; Cairns et al., 2011).

Similar to MYC, HIF1 is also a driver for metabolic switch from oxidative to glycolysis by upregulating glucose transporters and most glycolytic enzymes such as HK II, phosphofructokinase 1 (PFK1), fructose-bisphosphate aldolase A (ALDOA), ENO1, PKM2, and LDHA, as well as pentose phosphate pathway (PPP) enzymes (Iyer et al., 1998; Mathupala et al., 2001; Kim et al., 2006; Papandreou et al., 2006; Moldogazieva et al., 2020). In contrast to MYC, both HIF1 and HIF2 inhibit mitochondrial biogenesis in response to hypoxia. Under hypoxia, HIF1 promotes the transcription and activity of FOXO3a (Bakker et al., 2007), which represses a group of nuclear-encoded mitochondrial genes by directly antagonizing MYC on gene promoters (Jensen et al., 2011). Transcription of TFAM is facilitated by nuclear respiratory factor (NRF) interactions with peroxisome proliferator-activated receptor γ coactivator family members (PGC1α, PGC1β, and PRC1) in mitochondria biogenesis (Li et al., 2017). HIF1 also inhibits the transcription of PGC1β by antagonizing MYC (Zhang et al., 2007). A recent study shows that both HIF1α and HIF2α contribute to hypoxia-mediated inhibition of PGC1β and TFAM in human pulmonary endothelial cells (Zarrabi et al., 2017).

HIF1 directly transcriptionally activates PDK1 which inactivates pyruvate dehydrogenase (PDH), an enzyme converting mitochondrial pyruvate into acetyl coenzyme A, thus blocking pyruvate metabolism via the Krebs cycle (Kim et al., 2006; Papandreou et al., 2006). PDK1 not only reduces the mitochondrial oxygen consumption rate but also suppresses reactive oxygen species (ROS) production (Papandreou et al., 2006). HIF1 also fine-tunes hypoxic cell respiration by mediating switches in the subunit composition of cytochrome c oxidase from COX4-1 to COX4-2 (Fukuda et al., 2007). Furthermore, HIF1 reduces the overall mitochondrial mass by inducing mitochondrial autophagy. When cells are subjected to prolonged hypoxia, HIF1-dependent expression of BNIP3 protein triggers mitochondrial degradation through autophagy and stops the excessive production of mitochondrial ROS (Zhang et al., 2008). Such adaptive metabolic response may reduce energy consuming anabolic synthesis and prevent increased levels of ROS and cell death to increase cell survival during hypoxia.

Although HIF1α and MYC have opposing effects on mitochondrial function and biogenesis, they share common target genes on regulating glycolysis (Figure 2E) such as HK-II, PFK1, ENO1, LDHA. Most genes of glycolytic enzymes have MYC and HIF-1α DNA binding consensus sequences (Marbaniang and Kma, 2018). The relative expression levels of MYC and HIF1α proteins determine how they will interplay with each other during this process. It appears that when MYC is overexpressed, it overrides the inhibitory effects by HIF1α. HIF1α effects on MYC via binding to Max may be stoichiometrically diminished by increased MYC-Max complex formation. In Burkitt lymphoma, in which both MYC and HIF1α are highly expressed, HIF1α can actually collaborate with MYC to induce the expression of specific target genes, such as HK2, PDK1, and vascular endothelial growth factor (VEGF) (Kim et al., 2007) under hypoxia and confer resistance to cisplatin treatment (Nakajima et al., 2019). Similarly, high levels of N-MYC in N-MYC amplified neuroblastoma cells override HIF1α inhibition of cell cycle progression under hypoxia and cooperates with HIF1α to promote the expression of phosphoglycerate kinase 1 (PGK1), HK2, and LDHA (Qing et al., 2010). Ectopic expression of a stable form of HIF1 increases MYC-mediated tumorigenesis, while knockdown of HIF1α in B-cell lymphoma P493 cells suppresses its tumorigenesis (Gao et al., 2007). MYC overexpression can override the need for HIF1 for cell survival and propagation in response to hypoxia by inducing glutaminolysis and *de novo* lipogenesis (Munksgaard Thoren et al., 2017). For example, MYC and L-MYC amplified small cell lung carcinoma (SCLC) cells are dependent on glutamine, but not on glucose, for growth and survival (Munksgaard Thoren et al., 2017). Knockdown of HIF1α in these cells does not affect cell growth and cell survival at hypoxic conditions (Munksgaard Thoren et al., 2017). In addition to common targets regulated by both HIF-1α and HIF-2α, HIF-2α also stimulates the expression of specific genes involved in cell cycle progression, amino acid and lipid metabolism, angiogenesis and vasculature remodeling, tumor growth, metastasis, stemness as well as regulating tumor microenvironment (Covello et al., 2006; Keith et al., 2011; Elorza et al., 2012; Rankin and Giaccia, 2016; Hoefflin et al., 2020). Both MYC (Yue et al., 2017) and HIF-2α (Elorza et al., 2012) activate the expression of the amino acid transporter SLC7A5 to promote essential amino acid (EAA) uptake. Both MYC (Gouw et al., 2019; Casciano et al., 2020) and HIF-2α (Rankin et al., 2009; Qiu et al., 2015) regulate lipid metabolism.

MYC and HIFs also cooperate to promote tumor angiogenesis (Figure 2E). Under hypoxic conditions, HIF-1α can stimulate the expression of various pro-angiogenic factors, including VEGF, VEGF receptors FLT-1 and FLK-1, placental growth factor (PlGF), platelet-derived growth factor B (PDGF-B), plasminogen activator inhibitor-1 (PAI-1), the TIE-2 receptor, matrix metalloproteinases (MMP-2 and MMP-9) and angiopoietins (ANG-1 and ANG -2) (Hickey and Simon, 2006; Zimna and Kurpisz, 2015). Among all of these pro-angiogenic factors, VEGF is one of the most potent mediators of physiologic and pathological angiogenesis. Tumor angiogenesis can also be stimulated by MYC (Baudino et al., 2002). It was shown that oncogenic MYC cooperates with HIF1α to trigger VEGF production and secretion (Kim et al., 2007; Zhang et al., 2009). Although no conserved canonical MYC binding E box exists on VEGF promoter, MYC binds to the same genomic region where HIF1 binds (Kim et al., 2007). MYC also cooperates with HIF2α to promote tumor angiogenesis and hematogenous metastasis by transcriptional repression of miR-15-16 in hypoxia (Xue et al., 2015). miR-15-16 is an important negative regulator of fibroblast growth factor-2 (FGF2) which was proved to promote angiogenesis and metastasis (Cao et al., 2011).

The interplay of HIF family proteins with MYC plays an important role in tumorigenesis and progression. It has been shown that antioxidants such as N-acetylcycteine and vitamin C inhibit MYC-driven lymphoma xenograft growth *in vivo* by targeting HIF1, which can be rescued by the expression of oxygen-independent HIF1 mutant (Gao et al., 2007), supporting a key role of HIF1 in MYC-driven tumors. MYC activation in combination with *Vhl* and *Ink4a/Arf* deletion results in tumors in mice resembling human ccRCCs (Bailey et al., 2017). Interestingly, HIF-1α expression is frequently lost in ccRCCs correlating with poor patient survival (Monzon et al., 2011; Shen et al., 2011). Knockdown of HIF-1α promotes ccRCC cell proliferation and xenograft tumor growth (Shen et al., 2011). In VHL deficient ccRCCs with the expression of HIF-2α, but not HIF-1α, MYC activity is elevated together with the stimulation of cell cycle targets, cell proliferation and resistance to replication stress (Gordan et al., 2008). VHL^–^/HIF2α^+^ tumors that show more aggressiveness and proliferative capacity than VHL^–^/HIF1α^+^ tumors (Gordan et al., 2008). These data suggest that HIF-2α plays a major role in ccRCC initiation while HIF1a seems to play a role in inhibiting aggressive tumor behaviors. However, a recent study using *Vhl/p53/Rb1* deletion mouse model showed that HIF-1α is actually essential for ccRCC formation, whereas deletion of HIF-2α has moderate effects on tumor onset and growth but leads to increased intra-tumoral immune activation. Yet, deletion of HIF-1α in immortalized *Vhl/p53* null MEF cells increased cell proliferation, highlighting the role of HIF-1α in inhibiting proliferation of mouse *Vhl* deletion cells and tumor onset in the autochthonous setting. These seemingly contradicted findings by comparing the role of HIF-1α and HIF-2α in cell culture to that in xenograft models highlights the oncogenic role of HIF-1α in ccRCC initiation, the altered HIF-1α and HIF-2α balance in tumor development, contextual genetic background, and the role of HIF-2α in regulating tumor microenvironment. It would be interesting to further study the direct crosstalk of MYC with HIF-1α and HIF-2α in vivo in MYC activation in combination with *Vhl/Ink4a* null ccRCC model. Given the heterogeneity of tumor hypoxia, the expression of HIF-1α and HIF-2α may differ in different tumor areas. For example, compared to the cells in the tumor core, cells in the edge of tumors may exhibit chronic hypoxia, more stemness features, close interaction with tumor microenvironment and thus higher expression of HIF-2α (Mortezaee, 2020). HIF-1α can inhibit MYC to slow cell cycle progression under severe hypoxia condition whereas in mild hypoxia, HIF-2α may promote MYC activity by facilitating MYC-MAX dimerization.

Given the importance of the MYC-HIF interplay in cancer cells, it would be critical to further understand how MYC interplays with HIF in jointly regulating the expression of metabolic genes, mitochondria biogenesis, and the production of mitochondria intermediates for nucleotides, fatty acids, and bioamine biosynthesis. Does MYC set up the basal mitochondria activity while HIF shift the balance to glycolysis? The interplay may also heavily rely on the degree of oxygen deprivation and may differ at mild vs. severe hypoxia or acute vs. chronic hypoxia. There may also be different interplay in chromatin and mitochondria.

## Targeting MYC-HIF Crosstalk for Cancer Therapy

Given the essential roles of MYC and HIF in tumor progression and metastasis, there has been great clinical value in developing inhibitors targeting MYC and HIF as well as their regulators or downstream targets. Various approaches have been explored for targeting the MYC pathway (Whitfield et al., 2017). Although targeting MYC itself has often proven very challenging because of its nucleus localization and the absence of a deep surface-binding pocket, recent studies have shown that Omomyc, initially designed as a dominant-negative MYC peptide (Soucek et al., 1998, 2002) that competitively binds to E-box elements as heterodimer with MAX or homodimer and suppresses the binding of MYC to E-box (Beaulieu et al., 2019; Demma et al., 2019; Masso-Valles and Soucek, 2020), has cell-penetrating activity and the therapeutic potential in vivo in various cancer models with only mild and reversible side effects, demonstrating its potential in drug development for directly targeting MYC in cancer (Beaulieu et al., 2019; Wang et al., 2019; Masso-Valles and Soucek, 2020). Interestingly, Omomyc strongly inhibited the expression of a subset of genes directly regulated by HIF1α by reducing HIF1α binding to target promoters, thus inhibiting hypoxia-dependent glycolytic reprogramming and mitochondrial functionality in glioblastoma multiforme cells (Mongiardi et al., 2016). BET bromodomain inhibitors such as JQ1 downregulate MYC transcription by disrupting BRD4 binding at a distal MYC “super enhancer,” followed by genome-wide downregulation of Myc-dependent target genes (Delmore et al., 2011; Loven et al., 2013). Also, a number of MYC synthetic lethal pathways have been explored for targeting MYC-driven cancers (reviewed in Cermelli et al., 2014; Hsieh and Dang, 2016).

HIFs is also recognized as an attractive target for anticancer agents. But the complexity involved in the regulation of the HIF pathway has made developing specific HIF inhibitors very challenging too. Currently, there are no clinically approved HIF specific inhibitors. Yet, there are a number of molecules inhibiting HIF1 directly or indirectly, including targeting HIF1 transcription, translation, and protein degradation as well as targeting HIF-2α dimerization. Most of the reported HIF1 inhibitors were originally used for targeting other endogenous proteins and later they were found to inhibit HIF1 activity (Masoud and Li, 2015; Wigerup et al., 2016; Soni and Padwad, 2017). For example, EZN-2698, a synthetic antisense oligonucleotide, and Aminoflavone directly inhibit HIF1α mRNA expression, the PI3K/Akt/mTOR pathway inhibitors and topoisomerase I inhibitors suppress HIF1α translation, while HSP90 and HDAC inhibitors inhibit HIF1α degradation (Masoud and Li, 2015; Wigerup et al., 2016; Soni and Padwad, 2017). HIF1 inhibitor is thought to cooperate with anti-angiogenesis agents to overcome hypoxia-mediated therapy resistance. In addition, Acriflavine, a HIF1 inhibitor targeting HIF dimerization (Lee et al., 2009), inhibits chronic myelogenous leukemia cell growth *in vitro* and *in vivo*, which is partially associated with the reduction of MYC (Cheloni et al., 2017).

HIF-2α has recently emerged as a promising target in ccRCCs as it plays a major role in ccRCC tumorigenesis and progression. Following the discovery of the large internal cavity in the HIF-2α PAS-B domain that allows for ligand binding (Key et al., 2009; Scheuermann et al., 2009), several HIF-2α-specific antagonists have been discovered that disrupt the HIF-2α dimerization and show promising effects from pre-clinical to clinical trials, including PT2385, PT2399 and PT2977 (MK-6482) developed by Peloton Therapeutics. PT2399 was shown to effectively inhibit tumor growth in HIF-2α-high ccRCC cell lines and xenograft tumors (Chen et al., 2016; Cho et al., 2016). PT2385 also inhibited HIF-2α-driven gene expression and induced ccRCC tumor regression (Wallace et al., 2016). Recent result from a phase I clinic trial showed response, partial response or stable disease in ∼66% of patients with favorable safety profile and well tolerance for PT2385 (Courtney et al., 2018, 2020). Phase II clinic trials for PT2385 are ongoing in ccRCC (NCT03108066) and recurrent glioblastoma (NCT03216499). Preliminary results for phase II clinic trial for MK-6482 (PT2399) showed reduced size of target lesions in 86.9% (53/61) of pre-treated advanced ccRCC patients with 27.9% partial response rate (Eric Jonasch et al., 2020). A phase III clinic trial for MK-6482 in advanced ccRCC (NCT04195750) is currently in progress. If these clinic trials show promising efficacy, these HIF-2α inhibitors could be extended to other high grade and late stage solid tumors with high expression of HIF-2α.

As aerobic glycolysis and angiogenesis are common downstream effectors of MYC and HIF, which cooperate to drive the expression of many genes involved in both processes in cancer cells, targeting Warburg effect and pro-angiogenic factors have been of great interest. N-MYC amplified neuroblastoma cells are addicted to LDHA, which converts pyruvate to lactate. Knockdown of LDHA completely inhibits tumorigenesis *in vivo* and targeting LDHA could be a promising approach in treating neuroblastoma patients with N-MYC amplification (Qing et al., 2010). HKII is another potential therapeutic target to overcome cisplatin resistance in B-cell lymphoma (Nakajima et al., 2019). Inhibitors targeting metabolism or angiogenesis in combination with other chemotherapeutic drugs is an attractive strategy. For example, combination of apigenin and gefitinib, an epidermal growth factor receptor (EGFR) inhibitor, to treat EGFR-resistant mutant non-small cell lung cancers impairs energy utilization and suppresses cell growth and malignant behavior. They inhibit the activity of several oncogenic drivers such as MYC, HIF1α, and EGFR, reduce the protein expression of Gluts and MCT1, and inactivate the 5′ adenosine monophosphate-activated protein kinase (AMPK) signaling (Chen Z. et al., 2019). Together, targeting these downstream signaling pathways controlled by MYC-HIF crosstalk (Figure 2E) or directly co-targeting MYC and HIFs could emerge as effective therapeutics in advanced human cancers such as advanced ccRCCs.

## Conclusion and Perspectives

High MYC level was significantly associated with stabilized HIF1α expression in various cancers, such as prostate cancer, triple-negative breast cancer (TNBC) and animal model of CNS primitive neuroectodermal tumors (Malchenko et al., 2017; Boldrini et al., 2019; Cui and Jiang, 2019). Deregulated MYC cooperates with HIFs to regulate cancer cell adaptation to hypoxia, rewire metabolism, and promote angiogenesis. Also, high MYC and HIF expression is associated with poor outcome in various cancers such as prostate and breast cancers and clear cell renal cell carcinomas (Maroto et al., 2017; Boldrini et al., 2019; Cui and Jiang, 2019). Therefore, MYC and HIFs are potential biomarkers for targeting both pathways. The HIF-MYC interplay also plays a critical role in adaptive and innate immunity by regulating T cell development, activation, differentiation, metabolism and thus anti-tumor immunity (reviewed in Rankin and Giaccia, 2016; Gnanaprakasam et al., 2017). While HIF-1α deletion results in CD8^+^ T-cell infiltration, HIF-2α deletion leads to both CD4^+^ and CD8^+^ T-cell infiltration and activation as well as increased antigen presentation and interferon signaling in mouse ccRCC models, suggesting a role for HIF-2α in suppressing T-cell inflammation and intra-tumoral immune activation (Hoefflin et al., 2020). Therefore, it is important to further understand the role of MYC-HIF interplay in shaping tumor immune microenvironment and metastasis. Also, the MYC-HIF crosstalk may be dynamic and divergent in different type of tumors, various tumor stages, and hypoxia heterogeneity. As in other targeted therapy, therapeutic resistance is of a concern. For example, a gatekeeper mutation G323E in HIF-2α was identified to be responsible for PT2385 resistance after prolonged treatment (Courtney et al., 2020). Thus, further insights into the understanding of the MYC-HIF interplay are warranted for developing novel targeted therapeutics. It is also conceivable that the HIF-MYC axis further interplays with other oncogenic pathways such as ERK/MAPK, Akt/mTOR, WNT, and Notch signaling to alter cell metabolism, cell cycle, ribosome biogenesis, and genomic stability in tumorigenesis. Thus, the HIF-MYC targeted therapy may be in combination with inhibitors targeting these pathways as well.

## Author Contributions

YL, X-XS, DQ, and M-SD wrote and edited the manuscript. All authors contributed to the article and approved the submitted version.

## Conflict of Interest

The authors declare that the research was conducted in the absence of any commercial or financial relationships that could be construed as a potential conflict of interest.

## References

[B1] Arany Z. Huang L. E. Eckner R. Bhattacharya S. Jiang C. Goldberg M. A. (1996). An essential role for p300/CBP in the cellular response to hypoxia. *Proc. Natl. Acad. Sci. U.S.A.* 93 12969–12973. 10.1073/pnas.93.23.12969 8917528 PMC24030

[B2] Bailey S. T. Smith A. M. Kardos J. Wobker S. E. Wilson H. L. Krishnan B. (2017). MYC activation cooperates with Vhl and Ink4a/Arf loss to induce clear cell renal cell carcinoma. *Nat. Commun.* 8:15770. 10.1038/ncomms15770 PMC5472759 28593993

[B3] Bakker W. J. Harris I. S. Mak T. W. (2007). FOXO3a is activated in response to hypoxic stress and inhibits HIF1-induced apoptosis via regulation of CITED2. *Mol. Cell* 28 941–953. 10.1016/j.molcel.2007.10.035 18158893

[B4] Baluapuri A. Wolf E. Eilers M. (2020). Target gene-independent functions of MYC oncoproteins. *Nat. Rev. Mol. Cell Biol.* 21 255–267. 10.1038/s41580-020-0215-2 32071436 PMC7611238

[B5] Baudino T. A. McKay C. Pendeville-Samain H. Nilsson J. A. Maclean K. H. White E. L. (2002). c-Myc is essential for vasculogenesis and angiogenesis during development and tumor progression. *Genes Dev.* 16 2530–2543. 10.1101/gad.1024602 12368264 PMC187450

[B6] Beaulieu M. E. Jauset T. Masso-Valles D. Martinez-Martin S. Rahl P. Maltais L. (2019). Intrinsic cell-penetrating activity propels Omomyc from proof of concept to viable anti-MYC therapy. *Sci. Transl. Med.* 11:eaar5012, 10.1126/scitranslmed.aar5012 30894502 PMC6522349

[B7] Boldrini L. Bartoletti R. Giordano M. Manassero F. Selli C. Panichi M. (2019). C-MYC, HIF-1alpha, ERG, TKT, and GSTP1: an Axis in Prostate Cancer? *Pathol. Oncol. Res.* 25 1423–1429. 10.1007/s12253-018-0479-4 30357756

[B8] Brahimi-Horn M. C. Pouyssegur J. (2009). HIF at a glance. *J. Cell Sci.* 122 1055–1057. 10.1242/jcs.035022 19339544

[B9] Bretones G. Delgado M. D. Leon J. (2015). Myc and cell cycle control. *Biochim. Biophys. Acta* 1849 506–516. 24704206 10.1016/j.bbagrm.2014.03.013

[B10] Cairns R. A. Harris I. S. Mak T. W. (2011). Regulation of cancer cell metabolism. *Nat. Rev. Cancer* 11 85–95. 21258394 10.1038/nrc2981

[B11] Cantor J. R. Sabatini D. M. (2012). Cancer cell metabolism: one hallmark, many faces. *Cancer Discov.* 2 881–898. 10.1158/2159-8290.cd-12-0345 23009760 PMC3491070

[B12] Cao Y. Arbiser J. D’Amato R. J. D’Amore P. A. Ingber D. E. Kerbel R. (2011). Forty-year journey of angiogenesis translational research. *Sci. Transl. Med.* 3:114rv113. 10.1126/scitranslmed.3003149 PMC8265598 22190240

[B13] Casciano J. C. Perry C. Cohen-Nowak A. J. Miller K. D. Vande Voorde J. Zhang Q. (2020). MYC regulates fatty acid metabolism through a multigenic program in claudin-low triple negative breast cancer. *Br. J. Cancer* 122 868–884. 10.1038/s41416-019-0711-3 31942031 PMC7078291

[B14] Cermelli S. Jang I. S. Bernard B. Grandori C. (2014). Synthetic lethal screens as a means to understand and treat MYC-driven cancers. *Cold Spring Harb. Perspect. Med.* 4:a014209. 10.1101/cshperspect.a014209 24591535 PMC3935389

[B15] Cheloni G. Tanturli M. Tusa I. Ho DeSouza N. Shan Y. Gozzini A. (2017). Targeting chronic myeloid leukemia stem cells with the hypoxia-inducible factor inhibitor acriflavine. *Blood* 130 655–665. 10.1182/blood-2016-10-745588 28576876 PMC5942867

[B16] Chen C. Cai S. Wang G. Cao X. Yang X. Luo X. (2013). c-Myc enhances colon cancer cell-mediated angiogenesis through the regulation of HIF-1alpha. *Biochem. Biophys. Res. Commun.* 430 505–511. 10.1016/j.bbrc.2012.12.006 23237807

[B17] Chen W. Hill H. Christie A. Kim M. S. Holloman E. Pavia-Jimenez A. (2016). Targeting renal cell carcinoma with a HIF-2 antagonist. *Nature* 539 112–117. 27595394 10.1038/nature19796 PMC5340502

[B18] Chen Z. Tian D. Liao X. Zhang Y. Xiao J. Chen W. (2019). Apigenin combined with gefitinib blocks autophagy flux and induces apoptotic cell death through inhibition of HIF-1alpha, c-Myc, p-EGFR, and Glucose Metabolism in EGFR L858R+T790M-Mutated H1975 cells. *Front. Pharmacol.* 10:260. 10.3389/fphar.2019.00260 PMC6438929 30967777

[B19] Chen Y. Sun X. X. Sears R. C. Dai M. S. (2019). Writing and erasing MYC ubiquitination and SUMOylation. *Genes Dis.* 6 359–371. 10.1016/j.gendis.2019.05.006 31832515 PMC6889025

[B20] Cho H. Du X. Rizzi J. P. Liberzon E. Chakraborty A. A. Gao W. (2016). On-target efficacy of a HIF-2alpha antagonist in preclinical kidney cancer models. *Nature* 539 107–111. 10.1038/nature19795 27595393 PMC5499381

[B21] Compernolle V. Brusselmans K. Acker T. Hoet P. Tjwa M. Beck H. (2002). Loss of HIF-2alpha and inhibition of VEGF impair fetal lung maturation, whereas treatment with VEGF prevents fatal respiratory distress in premature mice. *Nat. Med.* 8 702–710. 10.1038/nm721 12053176

[B22] Compernolle V. Brusselmans K. Franco D. Moorman A. Dewerchin M. Collen D. (2003). Cardia bifida, defective heart development and abnormal neural crest migration in embryos lacking hypoxia-inducible factor-1alpha. *Cardiovasc. Res.* 60 569–579. 10.1016/j.cardiores.2003.07.003 14659802

[B23] Conacci-Sorrell M. McFerrin L. Eisenman R. N. (2014). An overview of MYC and its interactome. *Cold Spring Harb. Perspect. Med.* 4:a014357. 10.1101/cshperspect.a014357 24384812 PMC3869278

[B24] Corn P. G. Ricci M. S. Scata K. A. Arsham A. M. Simon M. C. Dicker D. T. (2005). Mxi1 is induced by hypoxia in a HIF-1-dependent manner and protects cells from c-Myc-induced apoptosis. *Cancer Biol. Ther.* 4 1285–1294. 10.4161/cbt.4.11.2299 16319523

[B25] Courtney K. D. Infante J. R. Lam E. T. Figlin R. A. Rini B. I. Brugarolas J. (2018). Phase I Dose-Escalation Trial of PT2385, a First-in-Class Hypoxia-Inducible Factor-2alpha Antagonist in Patients With Previously Treated Advanced Clear Cell Renal Cell Carcinoma. *J. Clin. Oncol.* 36 867–874. 10.1200/jco.2017.74.2627 29257710 PMC5946714

[B26] Courtney K. D. Ma Y. Diaz de Leon A. Christie A. Xie Z. Woolford L. (2020). HIF-2 Complex Dissociation, Target Inhibition, and Acquired Resistance with PT2385, a First-in-Class HIF-2 Inhibitor, in Patients with Clear Cell Renal Cell Carcinoma. *Clin. Cancer Res.* 26 793–803. 10.1158/1078-0432.ccr-19-1459 31727677 PMC7024660

[B27] Covello K. L. Kehler J. Yu H. Gordan J. D. Arsham A. M. Hu C. J. (2006). HIF-2alpha regulates Oct-4: effects of hypoxia on stem cell function, embryonic development, and tumor growth. *Genes Dev.* 20 557–570. 10.1101/gad.1399906 16510872 PMC1410808

[B28] Cowling V. H. Chandriani S. Whitfield M. L. Cole M. D. (2006). A conserved Myc protein domain, MBIV, regulates DNA binding, apoptosis, transformation, and G2 arrest. *Mol. Cell Biol.* 26 4226–4239. 10.1128/mcb.01959-05 16705173 PMC1489101

[B29] Cui J. Jiang H. (2019). Prediction of postoperative survival of triple-negative breast cancer based on nomogram model combined with expression of HIF-1alpha and c-myc. *Medicine* 98 e17370. 10.1097/md.0000000000017370 31577739 PMC6783179

[B30] Dang C. V. (2012a). Links between metabolism and cancer. *Genes Dev.* 26 877–890. 10.1101/gad.189365.112 22549953 PMC3347786

[B31] Dang C. V. (2012b). MYC on the path to cancer. *Cell* 149 22–35. 10.1016/j.cell.2012.03.003 22464321 PMC3345192

[B32] Dang C. V. Le A. Gao P. (2009). MYC-induced cancer cell energy metabolism and therapeutic opportunities. *Clin Cancer Res.* 15 6479–6483. 10.1158/1078-0432.ccr-09-0889 19861459 PMC2783410

[B33] Das B. Pal B. Bhuyan R. Li H. Sarma A. Gayan S. (2019). MYC Regulates the HIF2alpha Stemness Pathway via Nanog and Sox2 to Maintain Self-Renewal in Cancer Stem Cells versus Non-Stem Cancer Cells. *Cancer Res.* 79 4015–4025. 10.1158/0008-5472.can-18-2847 31266772 PMC6701948

[B34] David C. J. Chen M. Assanah M. Canoll P. Manley J. L. (2010). HnRNP proteins controlled by c-Myc deregulate pyruvate kinase mRNA splicing in cancer. *Nature* 463 364–368. 10.1038/nature08697 20010808 PMC2950088

[B35] Davis A. C. Wims M. Spotts G. D. Hann S. R. Bradley A. (1993). A null c-myc mutation causes lethality before 10.5 days of gestation in homozygotes and reduced fertility in heterozygous female mice. *Genes Dev.* 7 671–682. 10.1101/gad.7.4.671 8458579

[B36] Delmore J. E. Issa G. C. Lemieux M. E. Rahl P. B. Shi J. Jacobs H. M. (2011). BET bromodomain inhibition as a therapeutic strategy to target c-Myc. *Cell* 146 904–917. 21889194 10.1016/j.cell.2011.08.017 PMC3187920

[B37] Demma M. J. Mapelli C. Sun A. Bodea S. Ruprecht B. Javaid S. (2019). Omomyc reveals new mechanisms to inhibit the MYC Oncogene. *Mol. Cell Biol.* 39:e00248-19. 10.1128/MCB.00248-19 PMC6817756 31501275

[B38] Doe M. R. Ascano J. M. Kaur M. Cole M. D. (2012). Myc posttranscriptionally induces HIF1 protein and target gene expression in normal and cancer cells. *Cancer Res.* 72 949–957. 10.1158/0008-5472.can-11-2371 22186139 PMC3288382

[B39] Elorza A. Soro-Arnaiz I. Melendez-Rodriguez F. Rodriguez-Vaello V. Marsboom G. de Carcer G. (2012). HIF2alpha acts as an mTORC1 activator through the amino acid carrier SLC7A5. *Mol. Cell* 48 681–691. 10.1016/j.molcel.2012.09.017 23103253

[B40] Eric Jonasch F. D. Othon I. Wendy K. R. Vivek N. Benjamin L. M. Stephane O. (2020). Phase II study of the oral HIF-2α inhibitor MK-6482 for Von Hippel-Lindau disease–associated renal cell carcinoma. *J. Clin. Oncol.* 38:5003. 10.1200/jco.2020.38.15_suppl.5003 32753890

[B41] Farrell A. S. Sears R. C. (2014). MYC degradation. *Cold Spring Harb. Perspect. Med.* 4:a014365. 10.1101/cshperspect.a014365 24591536 PMC3935390

[B42] Florczyk U. Czauderna S. Stachurska A. Tertil M. Nowak W. Kozakowska M. (2011). Opposite effects of HIF-1alpha and HIF-2alpha on the regulation of IL-8 expression in endothelial cells. *Free Radic. Biol. Med.* 51 1882–1892. 10.1016/j.freeradbiomed.2011.08.023 21925595 PMC3202637

[B43] Fu R. Chen Y. Wang X. P. An T. Tao L. Zhou Y. X. (2016). Wogonin inhibits multiple myeloma-stimulated angiogenesis via c-Myc/VHL/HIF-1alpha signaling axis. *Oncotarget* 7 5715–5727. 10.18632/oncotarget.6796 26735336 PMC4868716

[B44] Fukuda R. Zhang H. Kim J. W. Shimoda L. Dang C. V. Semenza G. L. (2007). HIF-1 regulates cytochrome oxidase subunits to optimize efficiency of respiration in hypoxic cells. *Cell* 129 111–122. 10.1016/j.cell.2007.01.047 17418790

[B45] Gabay M. Li Y. Felsher D. W. (2014). MYC activation is a hallmark of cancer initiation and maintenance. *Cold Spring Harb. Perspect. Med.* 4:a014241. 10.1101/cshperspect.a014241 24890832 PMC4031954

[B46] Gao P. Tchernyshyov I. Chang T. C. Lee Y. S. Kita K. Ochi T. (2009). c-Myc suppression of miR-23a/b enhances mitochondrial glutaminase expression and glutamine metabolism. *Nature* 458 762–765. 10.1038/nature07823 19219026 PMC2729443

[B47] Gao P. Zhang H. Dinavahi R. Li F. Xiang Y. Raman V. (2007). HIF-dependent antitumorigenic effect of antioxidants in vivo. *Cancer Cell* 12 230–238. 10.1016/j.ccr.2007.08.004 17785204 PMC2084208

[B48] Gnanaprakasam J. N. R. Sherman J. W. Wang R. (2017). MYC and HIF in shaping immune response and immune metabolism. *Cytokine Growth Factor Rev.* 35 63–70. 10.1016/j.cytogfr.2017.03.004 28363691

[B49] Gordan J. D. Bertout J. A. Hu C. J. Diehl J. A. Simon M. C. (2007). HIF-2alpha promotes hypoxic cell proliferation by enhancing c-myc transcriptional activity. *Cancer Cell* 11 335–347. 10.1016/j.ccr.2007.02.006 17418410 PMC3145406

[B50] Gordan J. D. Lal P. Dondeti V. R. Letrero R. Parekh K. N. Oquendo C. E. (2008). HIF-alpha effects on c-Myc distinguish two subtypes of sporadic VHL-deficient clear cell renal carcinoma. *Cancer Cell* 14 435–446. 10.1016/j.ccr.2008.10.016 19061835 PMC2621440

[B51] Gouw A. M. Margulis K. Liu N. S. Raman S. J. Mancuso A. Toal G. G. (2019). The MYC oncogene cooperates with sterol-regulated element-binding protein to regulate lipogenesis essential for neoplastic growth. *Cell Metab.* 30:e555. 10.1016/j.cmet.2019.07.012 PMC6911354 31447321

[B52] Gruber M. Hu C. J. Johnson R. S. Brown E. J. Keith B. Simon M. C. (2007). Acute postnatal ablation of Hif-2alpha results in anemia. *Proc. Natl. Acad. Sci. U.S.A.* 104 2301–2306. 10.1073/pnas.0608382104 17284606 PMC1892942

[B53] Hickey M. M. Simon M. C. (2006). Regulation of angiogenesis by hypoxia and hypoxia-inducible factors. *Curr. Top. Dev. Biol.* 76 217–257. 10.1016/s0070-2153(06)76007-0 17118268

[B54] Hoefflin R. Harlander S. Schafer S. Metzger P. Kuo F. Schonenberger D. (2020). HIF-1alpha and HIF-2alpha differently regulate tumour development and inflammation of clear cell renal cell carcinoma in mice. *Nat. Commun.* 11:4111. 10.1038/s41467-020-17873-3 PMC7431415 32807776

[B55] Hsieh A. L. Dang C. V. (2016). MYC, metabolic synthetic lethality, and cancer. *Recent Results Cancer Res.* 207 73–91. 10.1007/978-3-319-42118-6_4 27557535

[B56] Hu C. J. Wang L. Y. Chodosh L. A. Keith B. Simon M. C. (2003). Differential roles of hypoxia-inducible factor 1alpha (HIF-1alpha) and HIF-2alpha in hypoxic gene regulation. *Mol. Cell Biol.* 23 9361–9374. 14645546 10.1128/MCB.23.24.9361-9374.2003 PMC309606

[B57] Ivan M. Kaelin W. G. Jr. (2017). The EGLN-HIF O2-sensing system: multiple inputs and feedbacks. *Mol. Cell* 66 772–779. 10.1016/j.molcel.2017.06.002 28622522 PMC5613951

[B58] Ivan M. Kondo K. Yang H. Kim W. Valiando J. Ohh M. (2001). HIFalpha targeted for VHL-mediated destruction by proline hydroxylation: implications for O2 sensing. *Science* 292 464–468. 10.1126/science.1059817 11292862

[B59] Iyer N. V. Kotch L. E. Agani F. Leung S. W. Laughner E. Wenger R. H. (1998). Cellular and developmental control of O2 homeostasis by hypoxia-inducible factor 1 alpha. *Genes Dev.* 12 149–162. 10.1101/gad.12.2.149 9436976 PMC316445

[B60] Jaakkola P. Mole D. R. Tian Y. M. Wilson M. I. Gielbert J. Gaskell S. J. (2001). Targeting of HIF-alpha to the von Hippel-Lindau ubiquitylation complex by O2-regulated prolyl hydroxylation. *Science* 292 468–472. 10.1126/science.1059796 11292861

[B61] Jensen K. S. Binderup T. Jensen K. T. Therkelsen I. Borup R. Nilsson E. (2011). FoxO3A promotes metabolic adaptation to hypoxia by antagonizing Myc function. *EMBO J.* 30 4554–4570. 10.1038/emboj.2011.323 21915097 PMC3243591

[B62] Keith B. Johnson R. S. Simon M. C. (2011). HIF1alpha and HIF2alpha: sibling rivalry in hypoxic tumour growth and progression. *Nat. Rev. Cancer* 12 9–22. 10.1038/nrc3183 22169972 PMC3401912

[B63] Key J. Scheuermann T. H. Anderson P. C. Daggett V. Gardner K. H. (2009). Principles of ligand binding within a completely buried cavity in HIF2alpha PAS-B. *J. Am. Chem. Soc.* 131 17647–17654. 10.1021/ja9073062 19950993 PMC2819816

[B64] Kim J. W. Gao P. Liu Y. C. Semenza G. L. Dang C. V. (2007). Hypoxia-inducible factor 1 and dysregulated c-Myc cooperatively induce vascular endothelial growth factor and metabolic switches hexokinase 2 and pyruvate dehydrogenase kinase 1. *Mol. Cell Biol.* 27 7381–7393. 10.1128/mcb.00440-07 17785433 PMC2169056

[B65] Kim J. W. Tchernyshyov I. Semenza G. L. Dang C. V. (2006). HIF-1-mediated expression of pyruvate dehydrogenase kinase: a metabolic switch required for cellular adaptation to hypoxia. *Cell Metab.* 3 177–185. 10.1016/j.cmet.2006.02.002 16517405

[B66] Kim J. W. Zeller K. I. Wang Y. Jegga A. G. Aronow B. J. O’Donnell K. A. (2004). Evaluation of myc E-box phylogenetic footprints in glycolytic genes by chromatin immunoprecipitation assays. *Mol. Cell Biol.* 24 5923–5936. 10.1128/mcb.24.13.5923-5936.2004 15199147 PMC480875

[B67] Koshiji M. Kageyama Y. Pete E. A. Horikawa I. Barrett J. C. Huang L. E. (2004). HIF-1alpha induces cell cycle arrest by functionally counteracting Myc. *EMBO J.* 23 1949–1956. 10.1038/sj.emboj.7600196 15071503 PMC404317

[B68] Koshiji M. To K. K. Hammer S. Kumamoto K. Harris A. L. Modrich P. (2005). HIF-1alpha induces genetic instability by transcriptionally downregulating MutSalpha expression. *Mol. Cell* 17 793–803. 10.1016/j.molcel.2005.02.015 15780936

[B69] Kress T. R. Sabo A. Amati B. (2015). MYC: connecting selective transcriptional control to global RNA production. *Nat. Rev. Cancer* 15 593–607. 10.1038/nrc3984 26383138

[B70] Kurland J. F. Tansey W. P. (2008). Myc-mediated transcriptional repression by recruitment of histone deacetylase. *Cancer Res.* 68 3624–3629. 10.1158/0008-5472.can-07-6552 18483244

[B71] Lando D. Peet D. J. Gorman J. J. Whelan D. A. Whitelaw M. L. Bruick R. K. (2002a). FIH-1 is an asparaginyl hydroxylase enzyme that regulates the transcriptional activity of hypoxia-inducible factor. *Genes Dev.* 16 1466–1471. 10.1101/gad.991402 12080085 PMC186346

[B72] Lando D. Peet D. J. Whelan D. A. Gorman J. J. Whitelaw M. L. (2002b). Asparagine hydroxylation of the HIF transactivation domain a hypoxic switch. *Science* 295 858–861. 10.1126/science.1068592 11823643

[B73] Lee K. Zhang H. Qian D. Z. Rey S. Liu J. O. Semenza G. L. (2009). Acriflavine inhibits HIF-1 dimerization, tumor growth, and vascularization. *Proc. Natl. Acad. Sci. U.S.A.* 106 17910–17915. 10.1073/pnas.0909353106 19805192 PMC2764905

[B74] Lee K. M. Giltnane J. M. Balko J. M. Schwarz L. J. Guerrero-Zotano A. L. Hutchinson K. E. (2017). MYC and MCL1 Cooperatively Promote Chemotherapy-Resistant Breast Cancer Stem Cells via Regulation of Mitochondrial Oxidative Phosphorylation. *Cell Metab.* 26 633–647 e637. 28978427 10.1016/j.cmet.2017.09.009 PMC5650077

[B75] Lee P. Chandel N. S. Simon M. C. (2020). Cellular adaptation to hypoxia through hypoxia inducible factors and beyond. *Nat. Rev. Mol. Cell Biol.* 21 268–283. 10.1038/s41580-020-0227-y 32144406 PMC7222024

[B76] Li F. Wang Y. Zeller K. I. Potter J. J. Wonsey D. R. O’Donnell K. A. (2005). Myc stimulates nuclearly encoded mitochondrial genes and mitochondrial biogenesis. *Mol. Cell Biol.* 25 6225–6234. 10.1128/mcb.25.14.6225-6234.2005 15988031 PMC1168798

[B77] Li P. A. Hou X. Hao S. (2017). Mitochondrial biogenesis in neurodegeneration. *J. Neurosci. Res.* 95 2025–2029. 10.1002/jnr.24042 28301064

[B78] Li Q. Kluz T. Sun H. Costa M. (2009). Mechanisms of c-myc degradation by nickel compounds and hypoxia. *PLoS One* 4:e8531. 10.1371/journal.pone.0008531 20046830 PMC2797325

[B79] Li Z. Van Calcar S. Qu C. Cavenee W. K. Zhang M. Q. Ren B. (2003). A global transcriptional regulatory role for c-Myc in Burkitt’s lymphoma cells. *Proc. Natl. Acad. Sci. U.S.A.* 100 8164–8169. 10.1073/pnas.1332764100 12808131 PMC166200

[B80] Loven J. Hoke H. A. Lin C. Y. Lau A. Orlando D. A. Vakoc C. R. (2013). Selective inhibition of tumor oncogenes by disruption of super-enhancers. *Cell* 153 320–334. 10.1016/j.cell.2013.03.036 23582323 PMC3760967

[B81] Lv X. Li J. Zhang C. Hu T. Li S. He S. (2017). The role of hypoxia-inducible factors in tumor angiogenesis and cell metabolism. *Genes Dis.* 4 19–24. 10.1016/j.gendis.2016.11.003 30258904 PMC6136595

[B82] Malchenko S. Sredni S. T. Bi Y. Margaryan N. V. Boyineni J. Mohanam I. (2017). Stabilization of HIF-1alpha and HIF-2alpha, up-regulation of MYCC and accumulation of stabilized p53 constitute hallmarks of CNS-PNET animal model. *PLoS One* 12:e0173106. 10.1371/journal.pone.0173106 28249000 PMC5332108

[B83] Marbaniang C. Kma L. (2018). Dysregulation of glucose metabolism by oncogenes and tumor suppressors in cancer cells. *Asian Pac. J. Cancer Prev.* 19 2377–2390. 30255690 10.22034/APJCP.2018.19.9.2377 PMC6249467

[B84] Markolovic S. Wilkins S. E. Schofield C. J. (2015). Protein Hydroxylation Catalyzed by 2-Oxoglutarate-dependent Oxygenases. *J. Biol. Chem.* 290 20712–20722. 10.1074/jbc.r115.662627 26152730 PMC4543633

[B85] Maroto P. Esteban E. Parra E. F. Mendez-Vidal M. J. Domenech M. Perez-Valderrama B. (2017). HIF pathway and c-Myc as biomarkers for response to sunitinib in metastatic clear-cell renal cell carcinoma. *Onco Targets Ther.* 10 4635–4643. 10.2147/ott.s137677 29033582 PMC5614781

[B86] Masoud G. N. Li W. (2015). HIF-1alpha pathway: role, regulation and intervention for cancer therapy. *Acta Pharm. Sin. B* 5 378–389. 10.1016/j.apsb.2015.05.007 26579469 PMC4629436

[B87] Masso-Valles D. Soucek L. (2020). Blocking Myc to treat cancer: reflecting on two decades of omomyc. *Cells* 9:883. 10.3390/cells9040883 32260326 PMC7226798

[B88] Mathupala S. P. Rempel A. Pedersen P. L. (2001). Glucose catabolism in cancer cells: identification and characterization of a marked activation response of the type II hexokinase gene to hypoxic conditions. *J. Biol. Chem.* 276 43407–43412. 10.1074/jbc.m108181200 11557773

[B89] Moldogazieva N. T. Mokhosoev I. M. Terentiev A. A. (2020). Metabolic heterogeneity of cancer cells: an interplay between HIF-1, GLUTs, and AMPK. *Cancers* 12:862. 10.3390/cancers12040862 32252351 PMC7226606

[B90] Mongiardi M. P. Savino M. Falchetti M. L. Illi B. Bozzo F. Valle C. (2016). c-MYC inhibition impairs hypoxia response in glioblastoma multiforme. *Oncotarget* 7 33257–33271. 10.18632/oncotarget.8921 27119353 PMC5078092

[B91] Monzon F. A. Alvarez K. Peterson L. Truong L. Amato R. J. Hernandez-McClain J. (2011). Chromosome 14q loss defines a molecular subtype of clear-cell renal cell carcinoma associated with poor prognosis. *Mod. Pathol.* 24 1470–1479. 10.1038/modpathol.2011.107 21725288 PMC4639322

[B92] Mortezaee K. (2020). Hypoxia induces core-to-edge transition of progressive tumoral cells: A critical review on differential yet corroborative roles for HIF-1alpha and HIF-2alpha. *Life Sci.* 242:117145. 10.1016/j.lfs.2019.117145 31816327

[B93] Munksgaard Thoren M. Vaapil M. Staaf J. Planck M. Johansson M. E. Mohlin S. (2017). Myc-induced glutaminolysis bypasses HIF-driven glycolysis in hypoxic small cell lung carcinoma cells. *Oncotarget* 8 48983–48995. 10.18632/oncotarget.16904 28430666 PMC5564742

[B94] Nakajima K. Kawashima I. Koshiisi M. Kumagai T. Suzuki M. Suzuki J. (2019). Glycolytic enzyme hexokinase II is a putative therapeutic target in B-cell malignant lymphoma. *Exp. Hematol.* 78:e43. 10.1016/j.exphem.2019.09.023 31560931

[B95] Nesbit C. E. Tersak J. M. Prochownik E. V. (1999). MYC oncogenes and human neoplastic disease. *Oncogene* 18 3004–3016. 10.1038/sj.onc.1202746 10378696

[B96] Oh E. T. Kim C. W. Kim H. G. Lee J. S. Park H. J. (2017). Brusatol-Mediated Inhibition of c-Myc Increases HIF-1alpha Degradation and Causes Cell Death in Colorectal Cancer under Hypoxia. *Theranostics* 7 3415–3431. 10.7150/thno.20861 28912885 PMC5596433

[B97] Osthus R. C. Shim H. Kim S. Li Q. Reddy R. Mukherjee M. (2000). Deregulation of glucose transporter 1 and glycolytic gene expression by c-Myc. *J. Biol. Chem.* 275 21797–21800. 10.1074/jbc.c000023200 10823814

[B98] Papandreou I. Cairns R. A. Fontana L. Lim A. L. Denko N. C. (2006). HIF-1 mediates adaptation to hypoxia by actively downregulating mitochondrial oxygen consumption. *Cell Metab.* 3 187–197. 10.1016/j.cmet.2006.01.012 16517406

[B99] Patten D. A. Lafleur V. N. Robitaille G. A. Chan D. A. Giaccia A. J. Richard D. E. (2010). Hypoxia-inducible factor-1 activation in nonhypoxic conditions: the essential role of mitochondrial-derived reactive oxygen species. *Mol. Biol. Cell* 21 3247–3257. 10.1091/mbc.e10-01-0025 20660157 PMC2938389

[B100] Peng J. Zhang L. Drysdale L. Fong G. H. (2000). The transcription factor EPAS-1/hypoxia-inducible factor 2alpha plays an important role in vascular remodeling. *Proc. Natl. Acad. Sci. U.S.A.* 97 8386–8391. 10.1073/pnas.140087397 10880563 PMC26957

[B101] Qing G. Skuli N. Mayes P. A. Pawel B. Martinez D. Maris J. M. (2010). Combinatorial regulation of neuroblastoma tumor progression by N-Myc and hypoxia inducible factor HIF-1alpha. *Cancer Res.* 70 10351–10361. 10.1158/0008-5472.can-10-0740 20961996 PMC3005134

[B102] Qiu B. Ackerman D. Sanchez D. J. Li B. Ochocki J. D. Grazioli A. (2015). HIF2alpha-Dependent Lipid Storage Promotes Endoplasmic Reticulum Homeostasis in Clear-Cell Renal Cell Carcinoma. *Cancer Discov.* 5 652–667. 10.1158/2159-8290.cd-14-1507 25829424 PMC4456212

[B103] Rahl P. B. Lin C. Y. Seila A. C. Flynn R. A. McCuine S. Burge C. B. (2010). c-Myc regulates transcriptional pause release. *Cell* 141 432–445. 10.1016/j.cell.2010.03.030 20434984 PMC2864022

[B104] Rankin E. B. Giaccia A. J. (2016). Hypoxic control of metastasis. *Science* 352 175–180. 10.1126/science.aaf4405 27124451 PMC4898055

[B105] Rankin E. B. Rha J. Selak M. A. Unger T. L. Keith B. Liu Q. (2009). Hypoxia-inducible factor 2 regulates hepatic lipid metabolism. *Mol. Cell Biol.* 29 4527–4538. 10.1128/mcb.00200-09 19528226 PMC2725738

[B106] Scheuermann T. H. Tomchick D. R. Machius M. Guo Y. Bruick R. K. Gardner K. H. (2009). Artificial ligand binding within the HIF2alpha PAS-B domain of the HIF2 transcription factor. *Proc. Natl. Acad. Sci.U.S.A.* 106 450–455. 10.1073/pnas.0808092106 19129502 PMC2626723

[B107] Seira N. Yamagata K. Fukushima K. Araki Y. Kurata N. Yanagisawa N. (2018). Cellular density-dependent increases in HIF-1alpha compete with c-Myc to down-regulate human EP4 receptor promoter activity through Sp-1-binding region. *Pharmacol. Res. Perspect.* 6 e00441. 10.1002/prp2.441 30455960 PMC6230926

[B108] Semenza G. L. (2003). Targeting HIF-1 for cancer therapy. *Nat. Rev. Cancer* 3 721–732. 10.1038/nrc1187 13130303

[B109] Semenza G. L. (2014). Oxygen sensing, hypoxia-inducible factors, and disease pathophysiology. *Annu. Rev. Pathol.* 9 47–71. 10.1146/annurev-pathol-012513-104720 23937437

[B110] Semenza G. L. (2020). The genomics and genetics of oxygen homeostasis. *Annu. Rev. Genomics Hum. Genet.* 21 183–204. 10.1146/annurev-genom-111119-073356 32255719

[B111] Semenza G. L. Jiang B. H. Leung S. W. Passantino R. Concordet J. P. Maire P. (1996). Hypoxia response elements in the aldolase A, enolase 1, and lactate dehydrogenase A gene promoters contain essential binding sites for hypoxia-inducible factor 1. *J. Biol. Chem.* 271 32529–32537. 10.1074/jbc.271.51.32529 8955077

[B112] Shen C. Beroukhim R. Schumacher S. E. Zhou J. Chang M. Signoretti S. (2011). Genetic and functional studies implicate HIF1alpha as a 14q kidney cancer suppressor gene. *Cancer Discov.* 1 222–235. 10.1158/2159-8290.cd-11-0098 22037472 PMC3202343

[B113] Shim H. Dolde C. Lewis B. C. Wu C. S. Dang G. Jungmann R. A. (1997). c-Myc transactivation of LDH-A: implications for tumor metabolism and growth. *Proc. Natl. Acad. Sci. U.S.A.* 94 6658–6663. 10.1073/pnas.94.13.6658 9192621 PMC21214

[B114] Soleymani Abyaneh H. Gupta N. Alshareef A. Gopal K. Lavasanifar A. Lai R. (2018). Hypoxia Induces the Acquisition of Cancer Stem-like Phenotype Via Upregulation and Activation of Signal Transducer and Activator of Transcription-3 (STAT3) in MDA-MB-231, a Triple Negative Breast Cancer Cell Line. *Cancer Microenviron.* 11 141–152. 10.1007/s12307-018-0218-0 30255421 PMC6250616

[B115] Soni S. Padwad Y. S. (2017). HIF-1 in cancer therapy: two decade long story of a transcription factor. *Acta Oncol.* 56 503–515. 10.1080/0284186x.2017.1301680 28358664

[B116] Soucek L. Helmer-Citterich M. Sacco A. Jucker R. Cesareni G. Nasi S. (1998). Design and properties of a Myc derivative that efficiently homodimerizes. *Oncogene* 17 2463–2472. 10.1038/sj.onc.1202199 9824157

[B117] Soucek L. Jucker R. Panacchia L. Ricordy R. Tato F. Nasi S. (2002). Omomyc, a potential Myc dominant negative, enhances Myc-induced apoptosis. *Cancer Res.* 62 3507–3510. 12067996

[B118] Stine Z. E. Walton Z. E. Altman B. J. Hsieh A. L. Dang C. V. (2015). MYC, Metabolism, and Cancer. *Cancer Discov.* 5 1024–1039. 26382145 10.1158/2159-8290.CD-15-0507 PMC4592441

[B119] Thomas L. R. Foshage A. M. Weissmiller A. M. Popay T. M. Grieb B. C. Qualls S. J. (2016). Interaction of MYC with host cell factor-1 is mediated by the evolutionarily conserved Myc box IV motif. *Oncogene* 35 3613–3618. 10.1038/onc.2015.416 26522729 PMC4853269

[B120] Thomas L. R. Wang Q. Grieb B. C. Phan J. Foshage A. M. Sun Q. (2015). Interaction with WDR5 promotes target gene recognition and tumorigenesis by MYC. *Mol. Cell* 58 440–452. 10.1016/j.molcel.2015.02.028 25818646 PMC4427524

[B121] Tian H. Hammer R. E. Matsumoto A. M. Russell D. W. McKnight S. L. (1998). The hypoxia-responsive transcription factor EPAS1 is essential for catecholamine homeostasis and protection against heart failure during embryonic development. *Genes Dev.* 12 3320–3324. 10.1101/gad.12.21.3320 9808618 PMC317225

[B122] To K. K. Sedelnikova O. A. Samons M. Bonner W. M. Huang L. E. (2006). The phosphorylation status of PAS-B distinguishes HIF-1alpha from HIF-2alpha in NBS1 repression. *EMBO J.* 25 4784–4794. 10.1038/sj.emboj.7601369 17024177 PMC1618093

[B123] Tu W. B. Helander S. Pilstal R. Hickman K. A. Lourenco C. Jurisica I. (2015). Myc and its interactors take shape. *Biochim. Biophys. Acta* 1849 469–483. 10.1016/j.bbagrm.2014.06.002 24933113

[B124] Vander Heiden M. G. Cantley L. C. Thompson C. B. (2009). Understanding the Warburg effect: the metabolic requirements of cell proliferation. *Science* 324 1029–1033. 10.1126/science.1160809 19460998 PMC2849637

[B125] Wallace E. M. Rizzi J. P. Han G. Wehn P. M. Cao Z. Du X. (2016). A Small-Molecule Antagonist of HIF2alpha Is Efficacious in Preclinical Models of Renal Cell Carcinoma. *Cancer Res.* 76 5491–5500. 10.1158/0008-5472.can-16-0473 27635045

[B126] Wang E. Sorolla A. Cunningham P. T. Bogdawa H. M. Beck S. Golden E. (2019). Tumor penetrating peptides inhibiting MYC as a potent targeted therapeutic strategy for triple-negative breast cancers. *Oncogene* 38 140–150. 10.1038/s41388-018-0421-y 30076412 PMC6318000

[B127] Wang G. L. Jiang B. H. Rue E. A. Semenza G. L. (1995). Hypoxia-inducible factor 1 is a basic-helix-loop-helix-PAS heterodimer regulated by cellular O2 tension. *Proc. Natl. Acad. Sci. U.S.A.* 92 5510–5514. 10.1073/pnas.92.12.5510 7539918 PMC41725

[B128] Wang V. Davis D. A. Haque M. Huang L. E. Yarchoan R. (2005). Differential gene up-regulation by hypoxia-inducible factor-1alpha and hypoxia-inducible factor-2alpha in HEK293T cells. *Cancer Res.* 65 3299–3306. 10.1158/0008-5472.can-04-4130 15833863

[B129] Weili Z. Zhikun L. Jianmin W. Qingbao T. (2019). Knockdown of USP28 enhances the radiosensitivity of esophageal cancer cells via the c-Myc/hypoxia-inducible factor-1 alpha pathway. *J. Cell Biochem.* 120 201–212. 10.1002/jcb.27305 30206969

[B130] Whitfield J. R. Beaulieu M. E. Soucek L. (2017). Strategies to inhibit myc and their clinical applicability. *Front. Cell Dev. Biol.* 5:10. 10.3389/fcell.2017.00010 PMC5322154 28280720

[B131] Wigerup C. Pahlman S. Bexell D. (2016). Therapeutic targeting of hypoxia and hypoxia-inducible factors in cancer. *Pharmacol. Ther.* 164 152–169. 10.1016/j.pharmthera.2016.04.009 27139518

[B132] Wise D. R. DeBerardinis R. J. Mancuso A. Sayed N. Zhang X. Y. Pfeiffer H. K. (2008). Myc regulates a transcriptional program that stimulates mitochondrial glutaminolysis and leads to glutamine addiction. *Proc. Natl. Acad. Sci. U.S.A.* 105 18782–18787. 10.1073/pnas.0810199105 19033189 PMC2596212

[B133] Wong W. J. Qiu B. Nakazawa M. S. Qing G. Simon M. C. (2013). MYC degradation under low O2 tension promotes survival by evading hypoxia-induced cell death. *Mol. Cell Biol.* 33 3494–3504. 10.1128/mcb.00853-12 23816886 PMC3753854

[B134] Xia X. Kung A. L. (2009). Preferential binding of HIF-1 to transcriptionally active loci determines cell-type specific response to hypoxia. *Genome Biol.* 10:R113. 10.1186/gb-2009-10-10-r113 PMC2784328 19828020

[B135] Xia X. Lemieux M. E. Li W. Carroll J. S. Brown M. Liu X. S. (2009). Integrative analysis of HIF binding and transactivation reveals its role in maintaining histone methylation homeostasis. *Proc. Natl. Acad. Sci. U.S.A.* 106 4260–4265. 10.1073/pnas.0810067106 19255431 PMC2657396

[B136] Xiao D. Ren P. Su H. Yue M. Xiu R. Hu Y. (2015). Myc promotes glutaminolysis in human neuroblastoma through direct activation of glutaminase 2. *Oncotarget* 6 40655–40666. 10.18632/oncotarget.5821 26528759 PMC4747359

[B137] Xue G. Yan H. L. Zhang Y. Hao L. Q. Zhu X. T. Mei Q. (2015). c-Myc-mediated repression of miR-15-16 in hypoxia is induced by increased HIF-2alpha and promotes tumor angiogenesis and metastasis by upregulating FGF2. *Oncogene* 34 1393–1406. 10.1038/onc.2014.82 24704828

[B138] Yoon D. Pastore Y. D. Divoky V. Liu E. Mlodnicka A. E. Rainey K. (2006). Hypoxia-inducible factor-1 deficiency results in dysregulated erythropoiesis signaling and iron homeostasis in mouse development. *J. Biol. Chem.* 281 25703–25711. 10.1074/jbc.m602329200 16787915

[B139] Yu F. White S. B. Zhao Q. Lee F. S. (2001). HIF-1alpha binding to VHL is regulated by stimulus-sensitive proline hydroxylation. *Proc. Natl. Acad. Sci. U.S.A.* 98 9630–9635. 10.1073/pnas.181341498 11504942 PMC55503

[B140] Yue M. Jiang J. Gao P. Liu H. Qing G. (2017). Oncogenic MYC Activates a Feedforward Regulatory Loop Promoting Essential Amino Acid Metabolism and Tumorigenesis. *Cell Rep.* 21 3819–3832. 10.1016/j.celrep.2017.12.002 29281830

[B141] Zarrabi A. J. Kao D. Nguyen D. T. Loscalzo J. Handy D. E. (2017). Hypoxia-induced suppression of c-Myc by HIF-2alpha in human pulmonary endothelial cells attenuates TFAM expression. *Cell Signal* 38 230–237. 10.1016/j.cellsig.2017.07.008 28709643 PMC5568858

[B142] Zhang H. Bosch-Marce M. Shimoda L. A. Tan Y. S. Baek J. H. Wesley J. B. (2008). Mitochondrial autophagy is an HIF-1-dependent adaptive metabolic response to hypoxia. *J. Biol. Chem.* 283 10892–10903. 10.1074/jbc.m800102200 18281291 PMC2447655

[B143] Zhang H. Gao P. Fukuda R. Kumar G. Krishnamachary B. Zeller K. I. (2007). HIF-1 inhibits mitochondrial biogenesis and cellular respiration in VHL-deficient renal cell carcinoma by repression of C-MYC activity. *Cancer Cell* 11 407–420. 10.1016/j.ccr.2007.04.001 17482131

[B144] Zhang J. Sattler M. Tonon G. Grabher C. Lababidi S. Zimmerhackl A. (2009). Targeting angiogenesis via a c-Myc/hypoxia-inducible factor-1alpha-dependent pathway in multiple myeloma. *Cancer Res.* 69 5082–5090. 10.1158/0008-5472.can-08-4603 19509231

[B145] Zhong H. De Marzo A. M. Laughner E. Lim M. Hilton D. A. Zagzag D. (1999). Overexpression of hypoxia-inducible factor 1alpha in common human cancers and their metastases. *Cancer Res.* 59 5830–5835. 10582706

[B146] Zimna A. Kurpisz M. (2015). Hypoxia-Inducible Factor-1 in Physiological and Pathophysiological Angiogenesis: Applications and Therapies. *Biomed. Res. Int.* 2015:549412. 10.1155/2015/549412 PMC4471260 26146622

